# Seroprevalence of Hepatitis Viruses B, C, D and HIV Infection Among Hemodialysis Patients in Kerman Province, South-East Iran

**DOI:** 10.5812/hepatmon.5969

**Published:** 2012-05-30

**Authors:** Mohammad Javad Zahedi, Sodaif Darvish Moghaddam, Seyed Moayed Alavian, Mahdieh Dalili

**Affiliations:** 1 Department of Internal Medicine, Physiology Research Center, Kerman University of Medical Sciences, Kerman, IR Iran; 2 Baqiyatallah Research Center for Gastroenterology and Liver diseases, Baqiyatallah University of Medical Sciences, Tehran, IR Iran; 3 Clinical Research Unit, Afzalipour Hospital, Kerman University of Medical Sciences, Kerman, IR Iran

**Keywords:** Hepatitis B Virus, Hepatitis C Virus, Hepatitis Delta Virus, Hemodialysis

## Abstract

**Background:**

The prevalence of hepatitis viruses in hemodialysis patients has been reported to be much greater than in the general population. Attention to local data, effectively guides health planners so that they can control infections and prevent nosocomial transmissions.

**Objectives:**

This cross sectional study was carried out to determine the prevalence of hepatitis B (HBV), hepatitis C (HCV), and hepatitis D (HDV) viruses, as well as the human immunodeﬁciency virus (HIV) in dialysis centers in the Kerman Province, in the southeast of Iran.

**Patients and Methods:**

All hemodialysis patients (n = 228) in 7 centers were enrolled in the study. Hepatitis B surface antigens (HBsAg), HCV antibodies (Ab), HDV Ab and HIV Ab were measured using speciﬁc enzyme linked immunoassay kits (ULTRA kit, bioMérieux, France) and conﬁrmed by a qualitative PCR assay.

**Results:**

The studied group was comprised of 92 (40.4%) females and 136 (59.6%) males. The mean age of the patients was 51 ± 9.5 years and the duration of hemodialysis was 39.7 ± 7.9 months. Positive HBsAg was found in 7% of cases, HCV Ab in 7%, and patients with both viruses were detected in 1.7% cases. HIV Ab and HDV Ab were negative in all cases. Out of the other risk factors, frequency of blood transfusions was signiﬁcantly correlated with positive HCV Ab (P < 0.008).

**Conclusions:**

Prevalence of HBV and HCV in hemodialysis patients was moderate to low in the Kerman Province, as in other parts of the country. Strict adherence to protective measures could lead to even lower rates.

## 1. Background

The use of hemodialysis for end stage renal disease (ESRD) has expanded increasingly in the past decades. Although this treatment modality has led to the increased longevity of patients; it also predisposes them to some infections, especially blood born viruses. Various studies have shown a higher prevalence of hepatitis B (HBV) and hepatitis C (HCV) viruses in hemodialysis patients than in the general population [1]. This may be explained by the sharing of dialysis machines, inappropriate preparation of parenteral drugs, and inadequate infection control methods in the hemodialysis units. Blood transfusion needs and the suppressed immunity of patients may also play a role [2]. Strict adherence to HBV infection control measures has led to a decline in HBV prevalence in hemodialysis units. These measures include; routine screening of donated blood products, separation of HBV dedicated dialysis machines, HBV vaccination and periodic measurement of anti–HBV antibodies [3].

Most patients infected with HCV are asymptomatic; therefore, HCV transmission may occur easily in the absence of standard infection control measures. It has been suggested that HCV transmission in dialysis units is mainly a result of environmental contamination, so dedicated machines are not currently recommended for HCV-positive patients. However, due to the lack of an effective vaccine, HCV is the most common cause of chronic viral hepatitis in dialysis units [4]. HBV and HCV infections in dialysis patients are important from many points of view; transmission of infection to other patients and/ or staff, progression of liver disease in pre-transplant or post-transplant immunosuppressed recipients, and an overall increase in morbidity and mortality rates [5][6]. Previous studies have determined an average prevalence of HBV and HCV in Iran, more speciﬁcally, HCV prevalence in 838 hemodialysis patients was found to be 13.2% in the Alavian study [7]. The prevalence of HBV in the hemodialysis patients in the Khuzestan Province has been reported to be 5.1% [8]. It seems that the prevalence of hepatitis virus infections is higher in developing countries than in developed countries. For example, the prevalence of HBV and HCV in Palestine has been reported to be 8.1% and 22% respectively [9]. In Yemen, the HCV prevalence rate was reported to be 62.7% [10], while in France it was 7.7% [11]. Focus on the prevalence of viral hepatitis in hemodialysis centers and the determination of their risk factors helps health planners in the country to operate more effectively, to reduce disease prevalence and recirculation, and ultimately reduce rates of morbidity and mortality.

## 2. Objectives

The aim of this study was to determine the prevalence of HBV, HCV, co-infection of these two viruses, hepatitis D virus (HDV), and human immunodeﬁciency virus (HIV) in hemodialysis centers in the Kerman Province, located in the southern part of Iran.

## 3. Patients and Methods

This cross sectional study was conducted in 6 cities and 7 hemodialysis centers in the Kerman Province in 2010. All of the 228 patients, who were under hemodialysis treatment, were included in the study. Demographic information and history of related risk factors were collected from the patients. Five mL of blood was drawn from each patient. HB surface antigens (HBsAg), HB core antibodies (HBcAb), HCV Ab and HIV Ab tests were performed on serum samples by speciﬁc enzyme linked immunoassay (ELISA) kits (ULTRA kit, bioMérieux, France). An HDV Ab (IgG) test was performed on the HBsAg positive serum samples. Qualitative PCR (AmpliSens, Russia for HBV and QIAGEN, Germany for HCV) was conducted on positive samples for HBsAg and anti-HCV Ab. In order to compare the frequency of risk factors, a sample of similar age and sex cases (n = 22) were selected from non-infected patients. All of the patients agreed to participate in the study.

### 3.1. Statistical Analysis

Statistical analysis was performed by SPSS version 16 (SPSS Inc, Chicago, IL, USA). Chi-square test and a Fischer’s exact test were used to compare the categorical variables and a Student’s t-test was used to compare continuous variables between the two groups. P < 0.05 was considered to be a statistical signiﬁcant level.

## 4. Results

The study was carried out on 228 chronic renal failure patients, which included 92 (40.4%) females and 136 (59.6%) males. The mean age of the patients was 51 ± 9.5 years old. Duration of hemodialysis was 39.7 ± 7.9 months. Out of these patients, 16 (7%) subjects including 8 females and 8 males had positive HBsAg. The mean age of these patients was 56 ± 14.3 years. Duration of dialysis at the time of the study was 29 ± 8 months. Reasons for renal failure were; diabetes mellitus in 6 (37.5%), hypertension in 4 (25%), polycystic kidney in 3 (18.8%) and unknown cause in 3 patients (18.8%). Predisposing factors for HBV infections were; blood transfusion with an average of 2 units in 9 (56.2%), ear piercing in 5 (31.2%), tattooing in 2 (12.5%), history of kidney transplant in 2 patients (12.5%) and phlebotomy in 1 patient (6.2%). A history of extramarital sexual behavior or intravenous drug usage were not found in the HBV infected patients. No signiﬁcant difference was observed in the risk factors above between the infected and non-infected patients (Table 1). Qualitative HBV PCR testing was positive in 6 (37.5%) of the positive HBV cases. Anti-HDV Ab was negative in all of the HBV infected patients.

**Table 1:** Comparison of Demographic Features and Risk Factors in Hemodialysis Patients With and Without HBV Infection

	**HBV Infected (n = 16)**	**Non-Infected (n = 22)**	***P* value**
Gender, No. (%)			0.78
Male	8 (50)	12 (54.5)	
Female	8 (50)	10 (45.5)	
Marital status, No. (%)			0.64
Single	1 (6.3)	2 (9.1)	
Married	12 (75)	16 (72.7)	
Divorced or separated	3 (18.7)	4 (18.2)	
Age of onset of dialysis, y, mean ±SD	14.3 ± 56.8	16.6 ± 51.4	0.29
Duration of dialysis, mo, mean ± SD	29 ± 8	38 ± 9	0.48
Tattoo, No. (%)			0.73
Yes	2 (12.5)	2 (9.1)	
No	14 (87.5)	20 (90.9)	
Phlebotomy, No. (%)			0.46
Yes	1 (6.2)	3 (13.6)	
No	15 (93.8)	19 (86.4)	
Extramarital sex, No. (%)			0.21
Yes	0 (0)	2 (9.1)	
No	16 (100)	20 (90.9)	
Drug injection, No. (%)			0.74
Yes	1 (6.2)	2 (9.1)	
No	15 (93.8)	20 (90.9)	
Blood transfusion , No. (%)			0.51
Yes	9 (56.2)	10 (45.5)	
No	7 (43.8)	12 (54.05)	

HCV Ab was positive in 16 patients (7%), comprising 12 males and 4 females with a mean age of 46 ± 4 years old. They had been under hemodialysis for a mean duration of 62.1 ± 6.5 months. HCV PCR was positive in 7 patients (43.8%). The main risk factor for HCV infection included blood transfusions in 14 patients (87.5 %) with an average of 2 units of blood. A signiﬁcant correlation was found between blood transfusion and positive HCV Ab (P < 0.008). Comparing other risk factors including previous kidney transplants, tattoos, and drug addiction, no signiﬁcant difference was found between the infected and non-infected patients (Table 2).

**Table 2:** Comparison of Demographic Features and Risk Factors in Hemodialysis Patients With and Without HCV Infection

	**HCV Infected (n = 16)**	**Non-Infected (n = 22)**	***P *value**
Gender, No. (%)			0.19
Male	12 (75)	12 (54.5)	
Female	4 (25)	10 (45.5)	
Marital status, No. (%)	5 (31.3)	2 (9.1)	0.64
Single	10 (62.5)	16 (72.7)	
Married	1 (6.2)	4 (18.2)	
Divorced or separated	46.9 ± 16.7	54.4 ± 16.6	
Age of onset of dialysis, y, mean ± SD	62.1 ± 6.5	45.8 ± 8.9	0.41
Duration of dialysis, mo, mean ± SD			0.18
Phlebotomy, No. (%)			0.12
Yes	0 (0)	3 (13.6)	
No	16 (100)	19 (86.4)	
Extramarital sex, No. (%)			0.21
Yes	0 (0)	2 (9.1)	
No	16 (100)	20 (90.9)	
Drug injection, No. (%)			0.73
Yes	2 (12.5)	2 (9.1)	
No	14 (87.5)	20 (90.9)	
Blood transfusion, No. (%)			0.008
Yes	14 (87.5)	10 (45.5)	
No	2 (12.5)	12 (54.5)	

Four patients (1.7%) had simultaneous positive HBsAg and anti-HCV Ab. HIV Ab was negative in all patients.

## 5. Discussion

The prevalence of viral hepatitis is greater in hemodialysis patients than in the general population and it affects their quality of life and mortality. The results of this study in the Kerman Province showed that the prevalence of HBV and HCV were 7% respectively and the rate of coinfection for both viruses was 1.7%. None of the patients were infected with HIV and/or hepatitis D. No signiﬁcant relationship was found between HBV and HCV infections with; age, sex, duration of dialysis, cause of renal failure, tattooing or previous history of kidney transplants. Only patients with positive HCV Ab were found to have a significant relationship with frequency of blood transfusions (P < 0.008). In this study, the rate of HCV infection in hemodialysis patients is comparable with those studies reported from other parts of the country. For example, in the Khuzestan Province, 7.9% of the hemodialysis patients had positive HCV Ab [8]. In another study by Alavian, the average rate of hepatitis C infections in hemodialysis patients in 12 provinces of the country has been estimated to be 7.6%. It has been concluded that Iran is among the low-prevalence countries in terms of HCV in hemodialysis patients [12]. It should be noted that the prevalence of the hepatitis C virus in the country’s general population is less than 1% [13]. So it suggests that in hemodialysis patients, the risk is increased several fold. Compared with other countries in the Middle East, the rate of hemodialysis patients’ infection with HCV has been reported as 28% in Jordan [14] and 48% in Syria [15], showing higher rates. Hepatitis B virus infection was also found in 7% of the hemodialysis patients in the Kerman Province which is similar to other studies in other areas of the country. For example, in the Khuzestan Province, 5.1% [8] and in Uromiye 6.5% [16] of the hemodialysis patients had positive HBsAg. The prevalence of HBsAg in the general population of Iran has been estimated to be 2.6% [17]. These ﬁndings also support the higher prevalence of HBV in hemodialysis patients. The role of occult hepatitis B infection in the spread of infection may need more attention. Despite negative HBsAg, these patients have positive HBV DNA and may infect others. As an example, in a study on 289 hemodialysis patients, it was determined that 50% of the patients who had isolated anti-HBC Ab were positive for HBV DNA [18]. In our study, 37.5% of HBsAg positive patients and 43% of HCV Ab positive patients had a positive qualitative PCR test. Positive HCV RNA PCR in hemodialysis patients in the Guilan Province was 50.8% [19]. A negative PCR might be due to intermittent viremia or low levels of virus in the serum. Although several risk factors for the acquisition of HCV and HBV infection in hemodialysis patients have been identiﬁed; in this study, only blood transfusion in HCV Ab positive patients showed a signiﬁcant relationship. This has been conﬁrmed in other studies as well [20]. However, nowadays, viral transmission via this route is limited due to more effective screening of donated blood [21]. In some studies, the duration of dialysis, prevalence of hepatitis C at the dialysis centers, and a previous history of kidney transplantation have been associated with the acquisition of the HCV [8][19][22]. In our study, no relationship was found between these risk factors and the acquisition of hepatitis viruses. Although the prevalence of hepatitis virus infections in hemodialysis units has been declining, a lack of attention to preventive measures has been demonstrated in various studies [23][24][25].

In conclusion, the prevalence of HBV and HCV infections in hemodialysis centers in the Kerman Province is moderate to low. Blood transfusion might be a risk factor for HCV acquisition. Strict adherence to protective measures could lead to signiﬁcantly lower rates.
